# Targeted Bottom–Up Mass Spectrometry Approach for the Relative Quantification of Post-Translational Modification of Bovine κ-Casein during Milk Fermentation

**DOI:** 10.3390/molecules27185834

**Published:** 2022-09-08

**Authors:** Sorel Tchewonpi Sagu, Harshadrai M. Rawel, Sascha Rohn

**Affiliations:** 1 Institute of Nutritional Science, University of Potsdam, Arthur-Scheunert-Allee 114-116, Nuthetal, 14558 Potsdam, Germany; 2 Institute of Food Technology and Food Chemistry, Technische Universität Berlin, Gustav-Meyer-Allee 25, 13355 Berlin, Germany

**Keywords:** bovine milk, fermentation, κ-casein, post-translational modifications, glycosylation, phosphorylation, mass spectrometry

## Abstract

κ-casein (κ-CN) is one of the key components in bovine milk, playing a unique role in the structuration of casein micelles. It contains in its chemical structure up to sixteen amino acid residues (mainly serine and threonine) susceptible to modifications, including glycosylation and phosphorylation, which may further be formed during milk processing. In this study, changes in post-translational modification (PTM) of κ-CN during bovine milk fermentation were investigated. One-to-five-day fermented milk samples were produced. A traditional bottom–up proteomics approach was used to establish a multiple-reaction monitoring (MRM) method for relative quantification of κ-CN PTM. Endoproteinase Glu-C was found to efficiently digest the κ-CN molecule. The developed LC-MS method was validated by performing assessments of linearity, precision, repeatability, reproducibility, limit of detection (LOD), and limit of quantification (LOQ). Among the yielded peptides, four of them containing serine and threonine residues were identified and the unmodified as well as the modified variants of each of them were relatively quantified. These peptides were (1) IPTINTIASGEPTSTTE _[140, 158]_, (2) STVATLE _[162, 168]_, (3) DSPE _[169, 172]_, and (4) INTVQVTSTAV _[180, 190]_. Distribution analysis between unmodified and modified peptides revealed that over 50% of κ-CN was found in one of its modified forms in milk. The fermentation process further significantly altered the composition between unmodified/modified κ-CN, with glycoslaytion being predominant compared to phosphorylation (*p* < 0.01). Further method development towards α and β-CN fractions and their PTM behavior would be an asset to better understand the changes undergone by milk proteins and the micellar structure during fermentation.

## 1. Introduction

Milk and dairy products such as yogurt and cheese are important sources of protein and peptides as well as many other nutrients [1]. Bovine milk dominates the world’s milk production with about 83% and production continues to increase progressively each year to meet the growing demand for dairy products [2]. Milk proteins play an important role in various milk processing technologies and in particular in the formation of acidic milk gel during fermentation [3]. Casein fraction, the main protein fraction of milk, representing about 80% of the total protein, is well known as the main contributor to structure formation in fermented milk products [4,5,6]. In contrast to human milk, bovine milk contains four caseins (CN): α_s1_-CN, α_s2_-CN, β-CN, and κ-CN [7,8]. Of these four, one of the key components of bovine milk is κ-CN, which plays a unique role in the structuration of the CN micelles. In fact, κ-CN is not affected by calcium and thus plays a key role in maintaining the stability and solubility of the micellar structure, as compared with the other CN [7]. κ-CN contains two cysteine residues in its structure that can form dimeric molecules. The amount of κ-CN distributed on the surface is responsible for the size of the micelle as well as other properties of the milk, including aggregation stability, which is a key parameter in milk processing [9].

The presence and nature of post-translational modifications (PTM) reflect the regulation of proteins, metabolic pathways, and chemical reactions in which they are involved. Milk proteins initially include many genetic variants and PTM such as glycosylation, phosphorylation, and disulfide bond formation, generating a large number of protein variants from a single gene product [10,11]. In fact, κ-CN constitutes the main glycoprotein in bovine milk [12]. Bijl et al. correlated the degree of glycosylation of κ-CN with micelle size; showing that a higher degree of glycosylation was associated with smaller micelles [13]. Moreover, it is now well established that changes in milk protein PTM including phosphorylation and glycosylation of threonine and serine residues may for instance affect the coagulation properties of milk [14,15,16]. κ-CN undergoes up to 16 documented PTM, including glycosylation of Thr142, Thr152, Ser153, Thr154, Thr157, Thr163, Ser170, and Thr186 residues, and phosphorylation of Ser148, Thr166, Ser170, and Ser187 residues [12,17,18]. Table S1 (supplementary data) presents a summary of key features, positions, and action descriptions of the different modifications experienced by the κ-CN.

Identification and quantification of PTM is therefore of particular interest here. Although considerable progress in this field has been recently documented, the development of accurate and robust proteomic methods remains a very challenging and complex issue [19]. Electrophoresis is one of the most widely used proteomics methods. Sodium dodecyl sulfate polyacrylamide gel electrophoresis (SDS-PAGE), for example, is generally applied for the separation of proteins based on their molecular weight, while two-dimensional gel electrophoresis separation is based on the difference in charge and molecular weight of proteins [11]. For identification and characterization purposes, the spots present on the gels after the separation are generally analyzed by mass spectrometry (MS). MS analysis of proteins has emerged as the key approach to tackle these challenges as the compounds involved can be identified, quantified, and characterized at the molecular level [20]. Depending on the form in which the proteins are analyzed, the type of separation technique, as well as the MS methodology involved, two different approaches are generally considered, namely top down and bottom up proteomics. The top–down approach consists of a direct analysis of intact proteins. The main advantage of this approach is that it allows a differentiation and identification of proteoforms and isoforms of proteins [21,22]. Here, one or two-dimensional liquid chromatography is generally used to separate the proteins with MS identification [19,23,24]. In contrast, the bottom–up approach consists in first digesting the proteins into small peptides with specific proteases prior to the MS analysis. The main advantages of this approach are the higher throughput and the higher sensitivity [25]. Bottom–up proteomics generally does not distinguish isoforms as well as protein similarities and thus similar peptides from different proteins are generally yielded. This may lead to inconsistent results, especially in label-free quantification using peptides [26]. The search for protein-specific peptides that can be used as biomarkers for identification and quantification is therefore an essential step. In the following, the peptides are easily separated and analyzed by MS/MS, allowing high reproducibility/robustness and thus comprehensive protein identification as well as quantification [19].

In this context, it can be hypothesized that PTM of milk proteins and especially κ-CN can be further developed during milk processing. Understanding and quantifying changes in κ-CN PTM occurring during the milk processing presents an interesting prospect for applications in monitoring and regulating the manufacturing parameters. Considering the mentioned background, the aim of this work was to develop a targeted based bottom–up MS analysis method to relatively quantify changes in κ-CN PTM during a model bovine milk fermentation process. For that purpose, milk and fermented milk samples after 1, 2, 3, 4, and 5 d were produced. Different digestive enzymes were investigated and finally, a highly specific and sensitive multiple-reaction monitoring (MRM) method was established and validated for quantification.

## 2. Results and Discussion

### 2.1. Method Development

A highly specific and sensitive MS method using MRM was established in order to investigate the PTM of milk κ-CN fraction during the fermentation process. Proteolytic digestion of proteins is a fundamental step for bottom–up proteomic analysis. Thus, the first step in the approach consisted in selecting the most appropriate protease to achieve optimal digestion. In silico tryptic digestion of κ-CN generated a large peptide TEIPTINTIASGEPTSTTEAVESTVATLEDSPEVIESPPEINTVQVTSTAV _[138, 190]_ with an initial mass of 5455.9 Da. This peptide also contained almost all the amino acid residues involved in PTM of κ-CN (Table S2, supplementary data). In fact, high-mass peptides and proteins are generally rather difficult to ionize, fragment, or detect [19]. As such large peptides are beyond the range of the filter for analysis by MS/MS systems (maximum *m/z* 1500), and considering the high number of possible modification combinations involved, tryptic digestion, although generally used as a conventional digestion enzyme, is therefore not successful. The absence of lysine and arginine residues in this portion of the κ-CN sequence limited the digestion efficiency of trypsin. Further investigations were conducted with different other enzymes and finally, endoproteinase Glu-C was found to be the most appropriate and was thus, selected to perform the digestion. Glu-C is a serine protease hydrolyzing protein chains on the C-terminal side of aspartic or glutamic acid residues in phosphate buffers while the enzymatic activity in ammonium-bicarbonate buffers is highly specific to glutamic acid [27,28]. Table S3 (supplementary data) shows the list of peptides obtained after the in silico digestion of κ-CN using Glu-C in ammonium bicarbonate buffer.

Among the peptides resulting from Glu-C digestion of κ-CN, four peptides were found containing one or more amino acid residues subject to PTM: (1) IPTINTIASGEPTSTTE _[140, 158]_ with six modified amino acid residues (glycosylation of Thr142, Thr152, Ser153, Thr154, and Thr157 and phosphorylation of Ser148); (2) STVATLE _[162, 168]_ with two modified amino acid residues (glycosylation of Thr163 and phosphorylation of Thr166); (3) DSPE _[169, 172]_ containing Ser170 undergoing two alternate modifications (glycosylation and phosphorylation), and (4) INTVQVTSTAV _[180, 190]_ having amino acid residues Thr186 and Ser187 subject to glycosylation and phosphorylation, respectively.

Peptide (1) containing six amino acid residues involved in modification offered up to 31 possible modified peptide combinations. These were initially tested and finally three of them were efficiently detected including IPTINTIASGEP**TST**PT**T**E _[140, 158]_, IPTINTIASGEP**TST**PTTE _[140, 158]_, and IPTINTIASGEPTS**T**PT**T**E _[140, 158]_ with the modified residues underlined. On the other hand, both modified variants of the peptide (3) D**S**PE _[169, 172]_ (phosphorylation and glycosylation of serine residue) and three modified variants of the peptide (2) S**T**VA**T**LE _[162, 168]_, S**T**VATLE _[162, 168]_, and STVA**T**LE _[162, 168]_) as well as peptide (4) INTVQV**TS**TAV _[180, 190]_, INTVQV**T**STAV _[180, 190]_, and INTVQVT**S**TAV _[180, 190]_ were successfully detected. The acquisition parameters for each precursor including the fragment voltage and collision energy were further individually optimized and based on the signal intensities, four transitions from the initial and modified peptides were selected for the final MRM method. Peptides, selected transitions, and their optimized detection parameters are presented in Table 1.

Glycosylation and phosphorylation led to a change in the binding affinity of the peptides. This was revealed through different retention times (RT) between the unmodified and modified peptides. For example, it was observed that peptide (1) IPTINTIASGEPTSTPTTE _[140, 158]_ showed a retention time of 11.26 min, while RT of 11.09, 10.44, and 10.26 min were recorded for its three modified forms, respectively (Table 1. Similarly, peptide (2) STVATLE _[162, 168]_ showed a RT of 9.58, while its modified form S**T**VA**T**LE _[162, 168]_ (glycosylation of Thr163 and phosphorylation of Thr166), retention time shifted to 7.62 min. In fact, the glycosylation and phosphorylation modifier groups appear to significantly affect the properties of the analyzed peptides compared to the intact protein peptides [29,30,31], making them more hydrophilic.

Moreover, optimum instrumental parameters were different between the unmodified peptide and its corresponding modified forms. For instance, the peptide INTVQVTSTAV _[180, 190]_ performed better for the fragment ions V [b6], V [y6], S [y4], and T [y5], while the modified peptide INTVQVT**S**TAV _[180, 190]_ with phosphorylation of Ser187 showed best signals for fragment ions V [b6], T [y5], N [b2], and Q [b5]. The optimal fragmentor voltages of 100 and 60 V, and collision energies of 10 and 9.8 eV were obtained for the unmodified and modified peptides, respectively (Table 1).

### 2.2. Method Validation

The developed MRM method was validated according to the guidelines of the European Medicines Agency (Note for guidance on validation of analytical procedures: text and methodology, CPMP/ICH/381/95). For this purpose, the parameters investigated were linearity, accuracy, repeatability (expressing the precision over a short interval of time/intra-day assay), reproducibility (inter-day assay), limit of detection (LOD), and limit of quantification (LOQ). The summary of the results for selected unmodified/modified peptides are presented in Table 2.

Linearity was assessed in the range of 0.6 to 8.0 µg equivalent injected protein. This was done by diluting the digested samples after the solid phase extraction (SPE) step to obtain the desired mass levels in an injection volume of 5 µL. The regression plots are presented in Figures S1–S3 (supplemental data). Coefficient of determination ranged from 0.9697 to 0.9990, demonstrating a strong correlation between the data in the selected interval of the analysis. The accuracy was evaluated by analyzing the recovery of the internal standard in water and sample matrices at three different concentration levels (experiments performed in triplicate). The results are presented in Figure S4 (supplemental data). No significant difference was observed between the different recovery values of the internal standard except for the milk matrix (*p* = 0.0218). The average recoveries of the internal standard of 93.1%; 96.8%; 100.6%; 104.5%; 98.0%, and 103.4% were found for milk and fermented milk samples after 1, 2, 3, 4, and 5 days, respectively. These results indicated that ion suppression/augmentation effect due to the matrices were not relevant.

Precision was also investigated by analyzing repeatability and reproducibility. Residual standard deviations given as a percentage (%RSD) were determined and the results presented in Table 2. Repeatability values ranged from 0.5 (INTVQVTSTAV) to 5.8% (S**T**VATLE _[162, 168]_) while reproducibility varied from 1.9 (internal standard) to 9.4% (S**T**VATLE _[162, 168]_). Detection limits were determined based on the standard deviation of the responses and the slopes, information which was collected from the linearity. LODs ranging from 0.269 to 1.864 µg equivalent injected protein, and LOQs from 0.895 to 6.212 µg equivalent injected protein were obtained, respectively.

### 2.3. Distribution of Unmodified and Modified Peptides in Milk Sample

**Figure 1:**
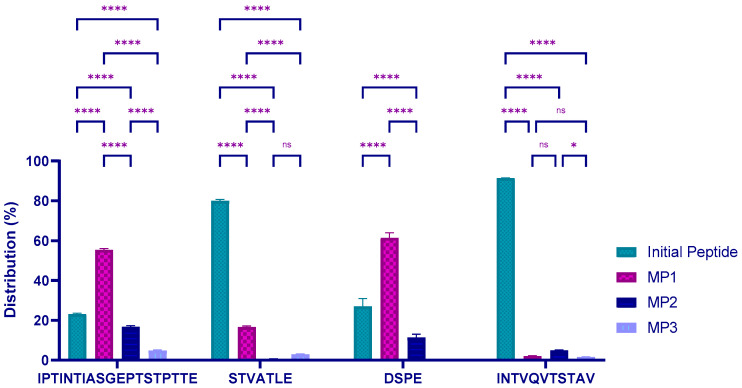
Distributions between the initial and corresponding modified peptides finally detected in the starting milk sample according to the developed LC-MS/MS method. With IPTINTIASGEP**TST**PT**T**E _[140, 158]_, IPTINTIASGEPTS**T**PT**T**E _[140, 158]_ and IPTINTIASGEP**TST**PTTE _[140, 158]_; S**T**VA**T**LE _[162, 168]_, STVA**T**LE _[162, 168]_ and S**T**VATLE _[162, 168]_; D**S**PE _[169, 172]_ and D**S**PE _[169, 172]_; and INTVQV**TS**TAV _[180, 190]_, INTVQVT**S**TAV _[180, 190]_ and INTVQV**T**STAV _[180, 190]_ corresponding to the modified forms MP1, MP2 and MP3 of peptides IPTINTIASGEPTSTPTTE _[140, 158]_, STVATLE _[162, 168]_, DSPE _[169, 172]_ and INTVQVTSTAV _[180, 190]_, respectively. Modified serine and threonine residues are underlined. Red and blue colors indicate glycosylation and phosphorylation modifications, respectively. Analyses were performed in triplicate (n = 3) and results presented as mean with standard deviations. Two-way ANOVA combined with multiple comparisons using Tukey statistical hypostasis tests were performed and *, and **** represent the confidence levels at *p* < 0.05 and 0.0001, respectively. ns = not significant.

The function and/or stability of proteins can be altered by specific structural modifications that occur on the peptide chain and the level of alteration can be related to the degree of protein modification [32]. Compositional analysis between unmodified and modified peptides in the initial unfermented milk sample was investigated and the results are presented in Figure 1. It was observed that peptide (1) IPTINTIASGEPTSTTE _[140, 158]_ accounted for 22%, while the three modified forms represented over 75%. MP1 (modified by glycosylation of T152, S153, T154, and T157) was significantly predominant (*p* < 0.0001) with 58% of the total sum of the unmodified and modified peptides. Similar results were observed with the peptide DSPE _[169, 172]_. Here, both modified forms were efficiently detected and the glycosylated (of serine residue) form represented up to 60% while the initial and phosphorylated forms constituted 27 and 13%, respectively (Figure 1). Conversely, the two other peptides analyzed showed a strong presence of their unmodified form from the digested milk sample compared with the detected modified forms. While the three possible modified forms accounted for less than 20%, the initial peptides STVATLE _[162, 168]_ and INTVQVTSTAV _[180, 190]_ represented 80% and 92%, respectively. Glycosylated peptides were found to be predominant in milk samples compared with phosphorylation. Finally, different modified peptide variants were detected along with their corresponding unmodified peptides. This suggested that a combination of unmodified and modified forms of κ-CN is present in the milk samples. Comparing different genetic variants, Bonfatti et al. observed no significant difference between κ-CN, indicating that the levels of glycosylation and phosphorylation are similar in different milk samples [33].

### 2.4. Changes in Post-Translational Modifications of κ-CN during Fermentation

**Figure 2:**
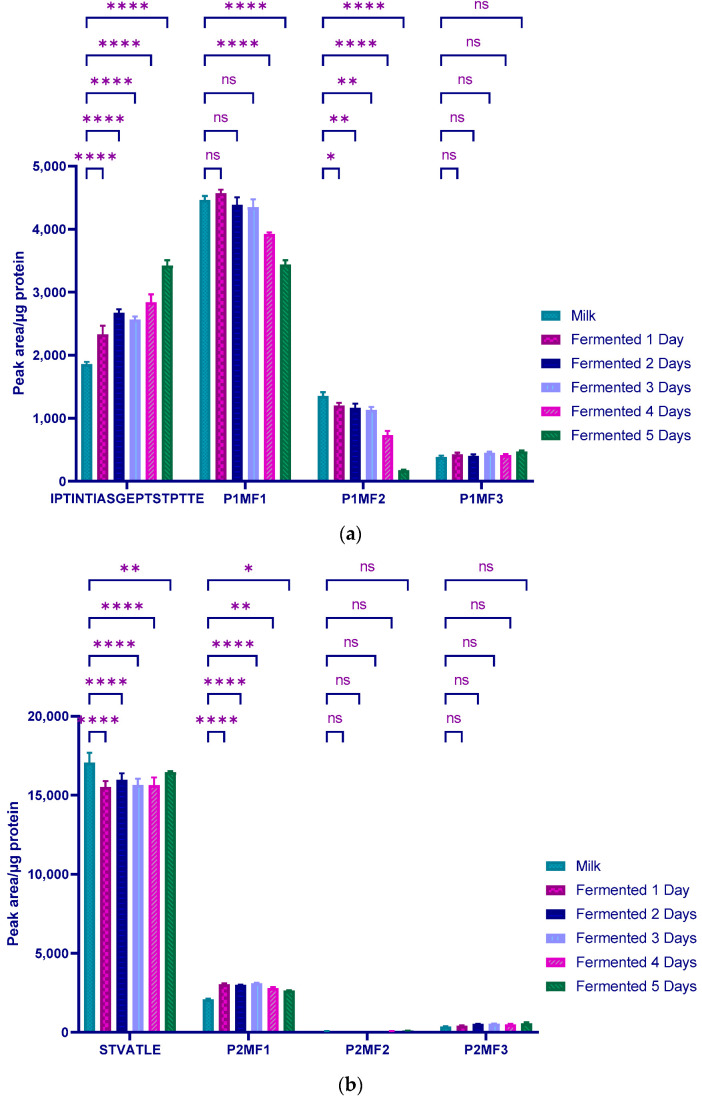
Changes in PTM of peptide (**a**) and (**b**) with fermentation time. P1MF1, P1MF2 and P1MF3 represent the modified peptides IPTINTIASGEP**TST**PT**T**E _[140, 158]_, IPTINTIASGEPTS**T**PT**T**E _[140, 158]_ and IPTINTIASGEP**TST**PTTE _[140, 158]_ while P2MF1, P2MF2 and P2MF3 represent modified peptide forms S**T**VA**T**LE _[162, 168]_, STVA**T**LE _[162, 168]_ and S**T**VATLE _[162, 168]_ of the peptides IPTINTIASGEPTSTPTTE a, respectively. Modified serine and threonine residues are underlined. **Red** and **blue** colors indicate glycosylation and phosphorylation modifications, respectively. Analyses were performed in triplicate (n = 3) and results presented as mean with standard deviations. Two-way ANOVA combined with multiple comparisons using Tukey statistical hypostasis tests were performed and *, ** and **** represent the confidence levels at *p* < 0.05; 0.01, and 0.0001, respectively. ns = not significant.

In order to evaluate the modifications occurring to the κ-CN structure during fermentation, different samples of milk and one-to-five-day fermented milk were analyzed under the conditions of the optimal conditions of the developed LC-ESI-MS/MS method. Figure 2 shows the relative abundance of peptides IPTINTIASGEPTSTTE _[140, 158]_ and STVATLE _[162, 168]_ and their respective modified forms during the fermentation. A significant increase (*p* < 0.0001) of the initial peptide IPTINTIASGEPTSTTE was observed _[140, 158]_ from the first day of fermentation. With the exception of the sample collected after 3 days of fermentation, the value gradually increased from 1859.4 ± 36.6 AP/µg protein (milk sample) to 3422.9 ± 85.3 after the fifth day of fermentation (Figure 2a). Conversely, the modified forms IPTINTIASGEP**TST**PT**T**E _[140, 158]_ (P1MF1; glycosylation of residues Thr152, Ser153, Thr154, and Thr157 and IPTINTIASGEPTS**T**PT**T**E _[140, 158]_ (P1MF2; glycosylation of residues Thr154 and Thr157) progressively decreased with fermentation. Analysis of variance revealed that this decrease in relative content was significant (*p* < 0.0001) after the fourth and fifth day of fermentation compared with the milk sample. The modified peptide P1MF1, which was initially predominant in the milk sample (4463.8 ± 64.7 PA/µg protein), representing almost two times the content of the initial peptide, and declined sharply to reach a level close to that of the initial peptide (3422.9 ± 85.3 and 3437.6 ± 70.6 PA/µg protein, respectively) at the end of the fifth day of fermentation (Figure 2a). However, the proportions of the peptide IPTINTIASGEP**TST**PTTE _[140, 158]_ (P1MF3; glycosylation of the residues of Thr152, Ser153, and Thr154), compared with the other structural forms, remained constant (around 400 PA/µg protein) in milk and fermented samples.

Fermentation induced a significant decrease (*p* < 0.001) of the native form of peptide STVATLE _[162, 168]_. However, this parent peptide, present in milk sample at 87% compared to the modified forms, remained predominant in the fermented samples. Its value dropped from 17,068 ± 619 to 15,513 ± 391 PA/µg protein after the first day of fermentation (Figure 2b). Subsequently, this value remained in the same range, with values of 15,980 ± 410, 15,649 ± 403, 15,641 ± 497, and 16451 ± 66 reported after 2, 3, 4, and 5 days of fermentation, respectively. On the other hand, the main modified peptide form STVATLE _[162, 168]_ (P2MF1) increased significantly with fermentation, rising from 2082 ± 48 to 3038 ± 63 PA/mg protein after the first day of fermentation. This value remained constant around 3000 PA/mg protein from day 2 to day 5 of fermentation (Figure 2b). The two other modified peptide forms STVATLE _[162, 168]_ (P2MF2) and STVATLE _[162, 168]_ (P2MF3) detected in the milk at proportions lower than 2% remained unaffected during the fermentation process, independently of the fermentation time (*p* > 0.05).

**Figure 3:**
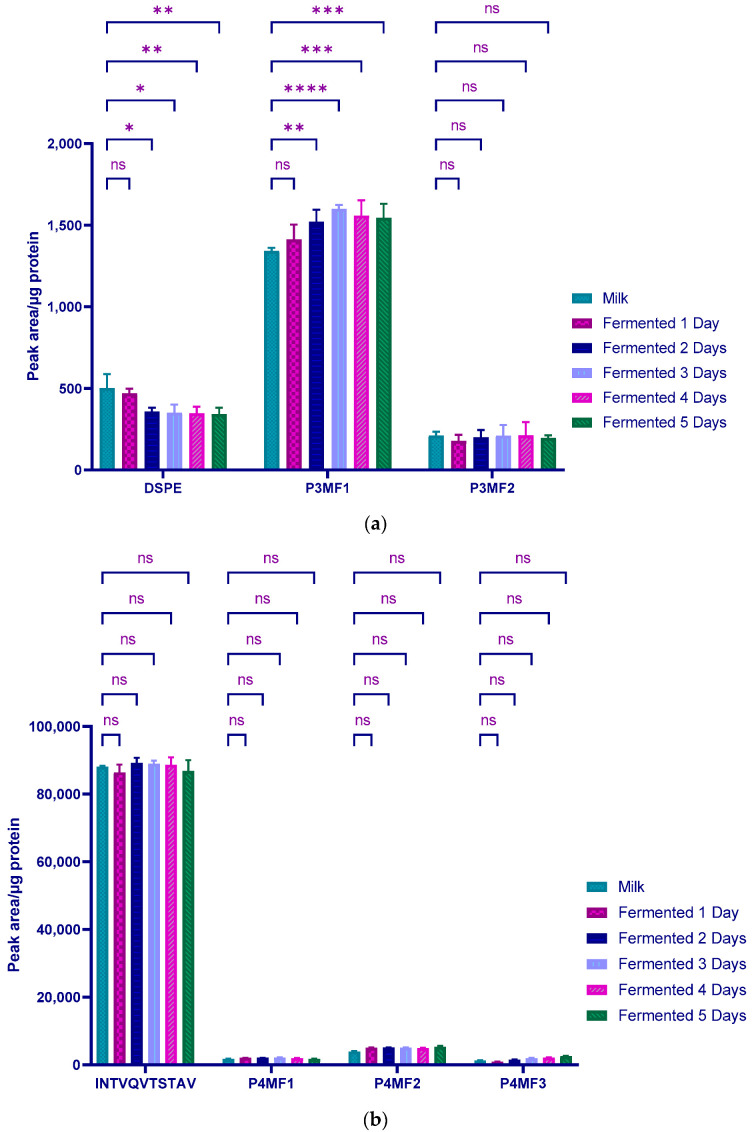
Changes in PTM of peptide (**a**) and (**b**) with fermentation time. MP1 and MP2 represent the modified peptides D**S**PE _[169, 172]_ and D**S**PE _[169, 172]_ of the peptide DSPE _[169, 172]_ while MP1, MP2 and MP3 are the modified peptides INTVQV**TS**TAV, INTVQVT**S**TAV and INTVQV**T**STAV of the peptides INTVQVTSTAV. Modified serine and threonine residues are underlined. **Red** and **blue** colors indicate glycosylation and phosphorylation modifications, respectively. Analyses were performed in triplicate (n = 3) and results presented as mean with standard deviations. *, **, ***, and **** represent the confidence levels at *p* < 0.05; 0.01; 0.001, and 0.0001, respectively. ns = not significant.

The peptide (3) DSPE _[169, 172]_ subjected to phosphorylation and glycosylation of Ser 170 was also investigated. The glycosylated variant (P3MF1) was found to be the predominant form representing overall over 58.1 ± 4.1% compared with the phosphorylated variant P3MF2 (11.0 ± 0.9%) and the native form of the peptide (27.2 ± 0.2%). With the fermentation process, there was a significant decrease in the relative amounts of the native peptide with the duration of fermentation starting on day 2 (*p* < 0.01). Then, the relative amounts of the DSPE _[169, 172]_ remained unchanged until the fifth day of fermentation. On the other hand, a significant increase of the relative amounts of the glycosylated form with the fermentation process, rising from 1341 ± 19 PA/µg protein in the milk sample to 1520 ± 91 PA/µg protein on the second day of fermentation (Figure 3a), was observed. Thereafter, the relative amounts of P3MF1 peptide remained constant until the fifth day of fermentation (with values of 1599 ± 25, 1557 ± 94, and 1544 ± 86 PA/µg protein reported between the third, fourth, and fifth days of fermentation, respectively). Statistical analysis of profiles related to the phosphorylated form of DSPE _[169, 172]_ (P3MF2) indicated that the relative quantities were not significantly different when comparing both milk and fermented samples. Values of 211 ± 23, 179 ± 37, 201 ± 42, 211 ± 65, 212 ± 81 and 197 ± 15 PA/µg protein were reported for milk and fermented samples after one, two, three, four and five days, respectively. It has been documented that up to 60% of the κ-CN can be glycosylated; the glycoside fraction being composed of N-acetylgalactosamine (GalNAc), galactose (Gal) and N-acetyl-neuraminic acid [14,34].

Comparison of the relative amounts of the peptide (4) INTVQVTSTAV and its modified variants before and after the fermentation process were also investigated and the results are presented in Figure 3b. This peptide involved three different types of PTM including a glycosylation of Thr186 (INTVQVTSTAV, P4MF2), phosphorylation of Ser187 (INTVQVTSTAV, P4MF3), and a peptide showing both modification (INTVQVTSTAV, P4MF1). Initial analysis showed that the unmodified peptide accounted for more than 91% of the relative amounts compared to the modified forms. This result remained relatively constant with fermentation. It can be seen from Figure 3b that there is no significant difference between the relative quantities obtained from the milk and 1 to 5 days fermented samples. This applied to the initial peptide as well as to the different modified forms. The fermentation process did not affect either Thr186 glycosylation or Ser187 phosphorylation. A possible assumption for this outcome is the peptide GFG being at the end of the κ-CN chain, it could be involved in other constellations so that Thr186 and Ser187 residues are not available to interact easily to be either glycosylated or phosphorylated. Fermentation, while altering the characteristics of the medium, was not sufficient to disturb this constellation to allow the both Thr186 and Ser187 residues to be more accessible for PTM.

### 2.5. Correlation between Modified Peptides Analyzed and κ-CN PTM

A conventional bottom–up targeted proteomics approach was used for relative quantification of the changes in PTM profiles of κ-CN during milk fermentation. As it is well known, it involves an indirect mode of investigation since the original protein undergoes hydrolysis to generate smaller peptides that are used as analytes for the subsequent mass spectrometry analysis. Digestion of milk and fermented samples with the enzyme GluC in ammonium bicarbonate buffer was performed. This yielded four peptides containing amino acid residues involved in κ-CN PTM including peptide (1) IPTINTIASGEPTSTPTTE _[140, 158]_, peptide (2) STVATLE _[162, 168]_, peptide (3) DSPE _[169, 172]_, and peptide (4) INTVQVTSTAV _[180, 190]_. The initial peptides and their modified variants showed different profiles depending on the samples analyzed. Peptides (2) and (4) were predominantly found in their native form while 77% of peptide (1) and 73% of peptide (3) were found to be modified. Considering peptide (3) in milk sample, for instance, it appeared that glycosylation was found to be predominant (61.5 ± 2.5%) compared with phosphorylation (11.4 ± 1.6%). Similarly, peptide (1) modified variant IPTINTIASGEP**TST**PT**T**E _[140, 158]_ and containing four glycosylated amino acid residues (Thr152, Ser153, Thr154, and Thr157) was found to be predominant (55.4 ± 0.7). This demonstrates that glycosylation appears to be more likely to be abundant compared with phosphorylation. Furthermore, while fermentation significantly impacted the relative content of peptides (1), (2), and (3) and their modified variants, this was not strong enough to drastically reverse the compositions in terms of ratio unmodified/modified peptides. Taking these results back to the protein level, two main conclusions can be extracted:

(1) The presence of both initial peptides and their modified variants shows that κ-CN in milk is found not only in its native form, but also in several other modified forms. The peptide (4) at the end of the chain (c-terminal side), which was mostly present in its original form, shows that this part of the κ-CN chain is however very slightly modified.

(2) The significant differences between the amounts of unmodified/modified peptides observed with the fermented samples in comparison to the milk sample suggest that fermentation significantly affects the PTM process of κ-CN (probably due to the change in pH of the medium and/or microbial metabolism). This could have an impact on the characteristics and especially the stability of the micelle structure of casein, of which κ-CN plays a key role.

**Figure 4:**
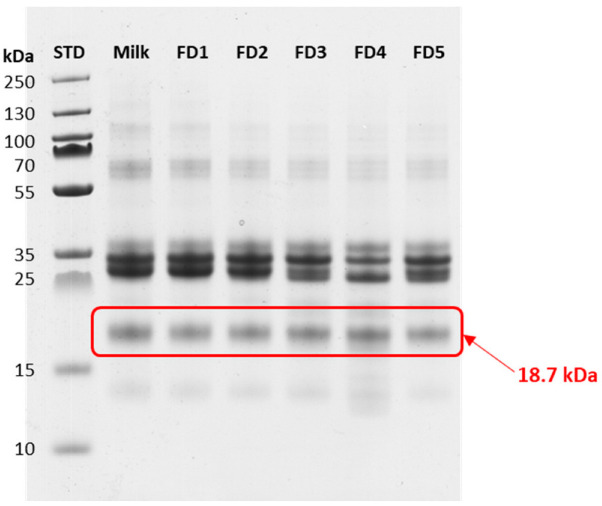
SDS PAGE of fermented milk samples. The experiment was performed under the reducing conditions. FD1, FD2, FD3, FD4, and FD5 represent the fermented samples after one, two, three, four, and five days, respectively.

Visualization of protein composition and specifically changes in κ-CN during fermentation was undertaken by performing SDS PAGE analyses. The tests were conducted under reduction conditions, and the results are presented in Figure 4. The analysis of the gel was processed with the software ImageLab version 6.1.0 build 7 (Bio-Rad Laboratories, inc., Hercules, CA, USA). This resulted in a distinctive band with a mass of 18.7 kDa in all samples analyzed that can be attributed to the κ-CN (18.9 kDa) and eventually beta-lactoglobulin (18.2 kDa). However, this is due to the fact that the glycosylation/phosphorylation of serine and threonine residues induces mass shifts (increase in mass compared with the initial protein masses) that are not significant enough to be observed by SDS-PAGE. Optionally, a more specific staining for the modified protein, e.g., for glycosylation would eventually reveal more relevant changes.

## 3. Materials and Methods

### 3.1. Materials and Chemicals

Commercial fresh milk with 1.5 and 3.5% fat (*w*/*v*) as well as the starter culture used for fermentation were purchased from a local supermarket (Potsdam, Germany). Ammonium bicarbonate, tris, and urea used as buffer for protein extraction were procured from Carl Roth GmbH (Karlsruhe, Germany). The sequencing grade endoproteinase Glu-C used to perform protein digestion was obtained from Roche Diagnostics GmbH, Mannheim, Germany. The nine amino acid residue peptide DPLNV(d8)LKPR used as internal standard for quantification purposes was provided by Peptide & elephants GmbH (Hennigsdorf, Germany). All other chemicals used in this work were of analytical grade.

### 3.2. Milk Fermentation

After heating at 80 °C for 20 min, milk samples were then cooled down to 37 °C. The fermentation process was performed by inoculating milk samples using 5% (*w*/*v*) of natural yogurt culture (with a ratio of 5 mg of starter per mL of milk). The mixtures were shortly stirred and incubated at 37 °C for one, two, three, four, and five days. The resulting pH of the fermented milks was about 4.05 to 5.10. To avoid changes in samples, milk as well as fermented samples were immediately frozen at −20 °C and then freeze-dried. Fermentation experiments were conducted in triplicate and the analysis were performed in random.

### 3.3. High-Resolution Mass Spectrometry Analysis

A targeted LC-MS/MS method was developed to evaluate the PTM behavior of milk κ-CN during the fermentation process. To this end, the approach used was similar to the method previously described, based on the Skyline software for the in-silico digestion as well as the development and optimization of mass spectrometry parameters for the analysis of the targeted modified peptides [35,36,37].

#### 3.3.1. Sample Preparation

20 mg of each lyophilized milk and fermented sample was dissolved in 1 mL of 100 mM ammonium bicarbonate buffer. The whole was homogenized at room temperature for 10 min under stirring conditions and then skimmed by centrifugation at 1000× *g* for 3 min, 4 °C. The skimmed phase was removed and 0.4 mL of the extracts was collected in the new microtubes. Samples were reduced at 50 °C for 15 min using 10 µL of 0.25 M Tris(2-carboxyethyl)phosphine hydrochloride (TCEP) solution and then alkylated for 15 min in the dark (by adding 10 µL of a solution of 0.25 M iodoacetamide—IAA). 135 µL of buffer (100 mM ammonium bicarbonate) and 20 µL of sequencing grade endoproteinase Glu-C were then added to the mixtures and the digestion was carried out for 20 h time at 37 °C under the shaking conditions. To stop the reaction, 15 µL of 40% formic acid was applied and after sonication for about 5 min, the samples were centrifuged at 7000× *g* for 5 min, and the solid phase extraction (SPE) was performed as a cleaning step.

The SPE step was performed on SPE bulk C18 Sorbens, (CHROMABOND C18 ec (Macherey-Nagel GmbH and CO. KG, Düren, Germany) as described previously. Briefly, columns containing 300 mg of C18 material were first activated with 6 mL of buffer A (50% acetonitrile and 50% bi-distilled water, 0.1% formic acid) and then conditioned with 6 mL of bi-distilled water. The digested samples were then loaded into the columns and washed with 6 mL of bi-distilled water. Finally, the analytes were recovered using 1 mL of buffer B consisting of 100% acetonitrile containing 0.1% formic acid. The collected samples were diluted in a 1:5 (*v*/*v*) ratio with distilled water and prior to the analysis, 100 µL of sample solution was mixed with 10 µL of the internal standard before being put in the vials.

#### 3.3.2. *In-Silico* Digestion

The κ-CN sequence (accession number: P02668) was first downloaded from the Uniprot online database as a FASTA file and then loaded into the Skyline software. The in-silico digestion was performed using Glu-C endoproteinase as enzyme in the presence of ammonium bicarbonate buffer, which under these conditions preferentially cleaves the C-terminal peptide bonds to glutamic acid residues, except when a proline residue was preceding. Other parameters set during this in-silico digestion were 0 as the maximum missed cleavage sites, filtering of peptides with a length of 3 to 25 amino acid residues, excluding from the analysis the amino acid residues at positions 1 to 21 which are related to the N-terminal signal peptide, and applying as structural modification the carbamidomethylation (C) of the cysteine residues. In order to further support the PTM, additional structural modifications (phosphorylation and glycosylation) were manually supplemented for each of the peptides containing threonine and serine residues subject to the modifications. Thus, the phosphorylation process led to an average additional charge of 79.87 (and a possible loss of 97.97-H_3_O_4_P during the fragmentation) while glycosylation (N-Acetylhexosamine) contributed to an increase in the mass of the precursors and fragments involved of 203.19. Both the original peptides and their modified variants were analyzed. Peptides containing several modified amino acid residues were initially selected and evaluated for different possible modification combinations. Precursors with charges ranging from +1 to +6 as well as y- and b-type ions with charge +1 were chosen for evaluation. The cut-off for the selection of precursors was 250–1500 *m/z*. Following the completion of the skyline project, it was submitted for analysis and further optimization of the LC-MS system operating parameters was performed.

#### 3.3.3. LC-MS/MS Conditions

The samples were analyzed on an Agilent Infinity 1260 HPLC system consisting of an auto-sampler vial, a multi-column thermostat equipped with a Kinetex C8 reversed phase chromatography column (2.6 µm, 100 A, 150 × 4.6 mm; Phenomenex, Torrance, CA, USA), and a binary pump (Agilent Technologies Sales & Services GmbH & Co.KG, Waldbronn, Germany). The detections were operated on an Agilent G6470A Triple Quad mass spectrometer detector coupled to an electrospray ionization source (ESI) parameterized in positive mode (Agilent Technologies Sales & Services GmbH & Co.KG, Waldbronn, Germany). Separation was performed over a column temperature of 30 °C and with a flow rate of 0.5 mL/min under gradient mode using 0.1% formic acid (eluent A) and 100% acetonitrile (eluent B) as mobile phases. The elution program was designed as follows: 0% eluent B from 0 to 1 min, 0–5% eluent B from 1 to 2 min, 5–50% eluent B from 2 to 16 min, 50–95% eluent B from 16 to 17 min, 95% eluent B from 17 to 20 min, 95–0% eluent B from 20 to 21 min, and 0% eluent B from 21 to 26 min. In addition, an equilibration time for the column of 4 min between each run (post run) with 0% eluent B was set. The desolvation gas temperature in the ionization source, a gas flow rate, a nebulizer pressure, and a dwell time of 275 °C, 11 L/min, 35 psi, and 20 ms were settled, respectively. Nitrogen used as the collision gas. Fragmentor voltage (in the range of 10–150 V) as well as collision energy (range of 5–50 ev) were individually optimized for each of the different precursor analyzed. Finally, a highly specific and sensitive multiple-reaction monitoring (MRM) method was established. The intensities of the 4 best product ions of each precursor (parent ions) were collected and summed to be used for quantification purposes.

### 3.4. Other Analysis

#### 3.4.1. Protein Content

The protein concentration of the extracts was measured according to the Bradford method [38] using a ready-to-use Bradford assay reagent (Pierce™ Coomassie Protein-Assay-Kit, the Protein-Assay-Kit, Thermo Fisher Scientific, Carlsbad, CA, USA) as described by the manufacturer and with bovine serum albumin (BSA) as calibration standard.

#### 3.4.2. Sodium Dodecyl Sulfate Polyacrylamide Gel Electrophoresis

The extracts were analyzed by sodium dodecyl sulfate-polyacrylamide gel electrophoresis (SDS-PAGE) according to Laemmli [39]. The assay was performed under denaturing conditions using a reducing agent to split the disulfide bonds. Briefly, 10 µL of the extracts was mixed with 10 µL of the sample buffer (NuPAGE LDS Sample Buffer 4X, Thermo Fisher). The mixture was heated to 95 °C for 5 min and then cooled to room temperature. 5 µL of each sample along with 5 µL of the broad range protein ladder (Thermo Scientific Spectra Multicolor, Size Range: 10 to 260 kDa) were loaded into the gels (NuPAGE™ 10%, Bis-Tris, 1.0 mm, Mini Protein Gel, 12-well; Thermo Scientific). The separation was performed at a constant current of 30 mA per gel for approximately 90 min, and the gels were then stained overnight using a Coomassie Brilliant Blue R-250 solution. After destaining for about 3 h with 10% acetic acid solution (the solution was changed regularly), the gels were scanned (Bio-500 Professional VIS Gel Scanner, SERVA Electrophoresis GmbH, Heidelberg, Germany) and densitometry analysis was performed to determine the relative concentrations of the casein fractions expressed in percent (ImageLab, Bio-Rad, Hemel Hempstead, UK).

### 3.5. Statistical Analysis

The fermentation experiments were performed in triplicate. Similarly, all the analyses were conducted in triplicate and the results were expressed as the mean ± standard deviation. The significance level of the results was assessed using the analysis of variance (ANOVA) followed by Fisher’s test (GraphPad Prism software Version 9.3, GraphPad Software LLC, San Diego, CA, USA). The results were significantly different for *p*-value < 0.05.

## 4. Conclusions

In this study, a bottom–up mass spectrometry approach was used to analyze the changes occurring in κ-CN PTMs during fermentation. Successful digestion of κ-CN molecules was achieved by using the endoproteinase GluC. This indicates that there is a need to have alternative, more specific proteases other than trypsin to successfully characterize certain molecular sequences of the proteins. The development in this field is rather limited, but the nowadays commercially available proline endopeptidase would be such a candidate, helpful in analysis of proline-rich plant proteins (e.g., cereals). Targeted LC-MS/MS analysis of the selected peptides is only possible when such enzyme specificities can be included in the preceding “in-silico” algorithms. Distribution analysis between unmodified and modified peptides revealed that the milk sample prior to fermentation already contained native κ-CN as well as different modified forms. The presented approach can therefore be integrated as a quality parameter for the raw minimally processed milk for determining its optimal usage. The fermentation process further significantly altered the composition between unmodified/modified κ-CN, glycosylation being predominant compared to phosphorylation. In that context, the further development of research towards other casein fractions and their PTM behavior during fermentation would be an asset to better understand the impact of this process on milk protein PTM. This also indicates much potential in integrating the presented approach while following the process initiated PTM and tailoring it to improve the quality of the end products.

## Figures and Tables

**Table 1:** Initial and corresponding PTM peptides selected in the positive ionization modus of the LC-MS/MS method.

Peptide No	Peptide Sequence	Precursor Ion	Charge State	RT (Min)	Product Ion	Type	Frag- Mentor (V)	Collision Energy (eV)
(1)	IPTINTIASGEPTSTPTTE _[140, 158]_	965.48621	2	11.26	1097.58372+	E [b11]	120	20.0
					833.38870+	P [y8]	120	20.0
					548.25623+	T [y5]	120	20.0
					447.20856+	P [y4]	120	20.0
	IPTINTIASGEPTSTPTTE _[140, 158]_	686.32612	4	11.09	650.28793+	P [y4]	140	20.0
					553.23516+	T [y3]	140	20.0
					312.19178+	T [b3]	140	20.0
					539.31877+	N [b5]	140	20.0
	IPTINTIASGEPTSTPTTE _[140, 158]_	508.64647	5	10.44	425.27585+	I [b4]	120	30.0
					350.15579+	T [y3]	120	30.0
					249.10811+	T [y2]	120	30.0
					312.19178+	T [b3]	120	30.0
	IPTINTIASGEPTSTPTTE _[140, 158]_	468.03060	5	10.26	1041.44701+	S [y6]	100	15.0
					640.36645+	T [b6]	100	15.0
					553.23516+	T [y3]	100	15.0
					539.31877+	N [b5]	100	15.0
(2)	STVATLE _[162, 168]_	720.37741	1	9.58	573.32425+	L [b6]	120	20.0
					460.24019+	T [b5]	120	20.0
					359.19251+	A [b4]	120	20.0
					362.19218+	T [y3]	120	20.0
	STVATLE _[162, 168]_	502.21520	2	7.62	743.28589+	T [b5]	100	20.0
					612.26404+	V [y5]	100	20.0
					261.14450+	L [y2]	100	20.0
					856.36996+	L [b6]	100	20.0
	STVATLE _[162, 168]_	462.23203	2	10.66	562.27188+	A [b4]	160	20.0
					433.22929+	A [y4]	160	20.0
					261.14450+	L [y2]	160	20.0
					491.23477+	V [b3]	160	20.0
	STVATLE _[162, 168]_	800.34374	1	10.27	713.31171+	T [y6]	140	40.0
					513.19562+	A [y4]	140	40.0
					359.19251+	A [b4]	140	40.0
					540.20652+	T [b5]	140	40.0
(3)	DSPE _[169, 172]_	447.17217	1	6.24	315.11868+	S [z3]	20	25.0
					228.08665+	P [z2]	20	25.0
					203.06625+	S [b2]	20	25.0
					245.11320+	P [y2]	20	25.0
	DSPE _[169, 172]_	325.62941	2	7.09	520.22493+	P [c3]	80	10.0
					268.11594++	S [y3]	80	10.0
					245.11320+	P [y2]	80	10.0
					535.22460+	S [y3]	80	10.0
	DSPE _[169, 172]_	527.13850	1	7.51	283.03258+	S [b2]	80	25.0
					271.09246+	P [x2]	80	25.0
					245.11320+	P [y2]	80	25.0
					395.08501+	S [z3]	80	25.0
(4)	INTVQVTSTAV _[180, 190]_	566.81405	2	10.39	655.37735+	V [b6]	100	10.0
					577.31917+	V [y6]	100	10.0
					377.20308+	S [y4]	100	10.0
					478.25075+	T [y5]	100	10.0
	INTVQVTSTAV _[180, 190]_	708.33691	2	9.99	655.37735+	V [b6]	120	23.0
					329.18195+	T [b3]	120	23.0
					228.13427+	N [b2]	120	23.0
					761.29646+	T [y5]	120	23.0
	INTVQVTSTAV _[180, 190]_	334.68051	4	9.57	655.37735+	V [b6]	80	10.0
					329.18195+	T [b3]	80	10.0
					290.17105+	T [y3]	80	10.0
					556.30894+	Q [b5]	80	10.0
	INTVQVTSTAV _[180, 190]_	404.86724	3	7.89	655.37735+	V [b6]	60	9.8
					558.21709+	T [y5]	60	9.8
					228.13427+	N [b2]	60	9.8
					556.30894+	Q [b5]	60	9.8

RT is the retention time. Modified serine and threonine residues are underlined. **Red** and **blue** colors indicate glycosylation and phosphorylation modifications, respectively.

**Table 2:** Summary of method validation with selected initial and modified peptides.

	Linearity (R Squared)	Repeatability (% RSD)	Reproducibility (% RSD)	LOD (ng Protein)	LOQ (ng Protein)
DPLNV(d8)LKPR (IS)	0.9902	0.751	1.904	-	-
IPTINTIASGEPTSTPTTE _[140, 158]_	0.9849	1.970	2.992	1.047	3.491
IPTINTIASGEPTSTPTTE _[140, 158]_	0.9924	1.450	5.194	0.838	2.793
IPTINTIASGEPTSTPTTE _[140, 158]_	0.9796	4.587	5.990	1.374	4.579
IPTINTIASGEPTSTPTTE _[140, 158]_	0.9818	3.909	7.504	1.280	4.265
STVATLE _[162, 168]_	0.9990	3.168	4.134	0.269	0.895
STVATLE _[162, 168]_	0.9830	2.341	5.430	0.997	3.323
STVATLE _[162, 168]_	0.9772	5.776	9.392	1.400	4.665
DSPE _[169, 172]_	0.9754	1.724	6.476	1.309	4.362
INTVQVTSTAV _[180, 190]_	0.9889	0.475	4.694	1.089	3.629
INTVQVTSTAV _[180, 190]_	0.9697	3.303	5.318	1.864	6.212

IS = internal standard. RSD = relative standard deviation, LOD & LOQ = limits of detection & quantification. Modified serine and threonine residues are underlined. **Red** and **blue** colors indicate glycosylation and phosphorylation modifications, respectively.

## Data Availability

Not applicable.

## Supplementary Materials

The following supporting information can be downloaded at: https://www.mdpi.com/article/10.3390/molecules27185834/s1, Figure S1. Linearity of the peptide IPTINTIASGEPTSTPTTE using the developed LC-MS/MS method; Figure S2. Linearity of the peptide INTVQVTSTAV; Figure S3. Linearity of the peptide STVATLE; Figure S4. Recovery of the injected internal standards DPLNV(d8)LKPR in the different milk and fermented milk matrixes; Table S1. List of amino acid modifications (PTM) of bovine κ-CN according to the online database UniProtKB; Table S2. List of peptides obtained after the in-silico tryptic digestion of κ-CN using the PeptideCutter tool of UniprotKB; Table S3. List of peptides obtained after the in-silico GluC-E digestion (Ambi buffer) of κ-CN.
