# Supplementary material for: Targeted Bottom–Up Mass Spectrometry Approach for the Relative Quantification of Post-Translational Modification of Bovine κ-Casein during Milk Fermentation
Source: Molecules. 2022 Sep 8;27(18):5834. doi: 10.3390/molecules27185834 (PMC9506521; doi:10.3390/molecules27185834)
Supplement: Supplementary file 1 [file molecules-27-05834-s001.zip › molecules-1874083-supplementary.pdf]

Supplementary Data

Targeted Bottom-Up Mass Spectrometry Approach for the Relative Quantification of Post Translational Modification of Bovine κ-Casein during Milk Fermentation

Sorel Tchewonpi Sagu [1,2], Harshadrai M. Rawel [1] and Sascha Rohn [2,*]

[1] Institute of Nutritional Science, University of Potsdam, Arthur-Scheunert-Allee 114-116, Nuthetal, 14558 Potsdam, Germany

[2] Institute of Food Technology and Food Chemistry, Technische Universität Berlin, Gustav-Meyer-Allee 25, 13355 Berlin, Germany

* Correspondence: rohn@tu-berlin.de; Tel.: +49-30-314-72583

Content

Figure S1. Linearity of the peptide (a) E.IPTINTIASGEPTSTPTTE.A [140, 158] and it modified forms (b) E.IPTINTIASGEPTSTPTTE.A [140, 158]; (c) E.IPTINTIASGEPTSTPTTE.A [140, 158] and (d) E.IPTINTIASGEPTSTPTTE.A [140, 158] using the developed LC-MS/MS method. Red and green colors represent glycosylated and phosphorylated amino acid residues, respectively.

Figure S2. Linearity of the peptide (a) E.INTVQVTSTAV and it modified form (b) E.INTVQVTSTAV using the developed LC-MS/MS method. Red and green colors represent glycosylated and phosphorylated amino acid residues, respectively.

Figure S3. Linearity of the peptide (a) E.STVATLE.D [162, 168] and it modified forms (b) E.STVATLE.D [162, 168] and (d) E.STVATLE.D [162, 168] using the developed LC-MS/MS method. Red and green colors represent glycosylated and phosphorylated amino acid residues, respectively.

Figure S4. Recovery of the injected internal standards DPLNV(d8)LKPR in the different milk and fermented milk matrixes. The experiments were performed in triplicate and the results are expressed as percent (%) comparing to the internal standard in distilled water (n=3). FM-1, FM-2, FM-3, FM-4 and FM-5 are the fermented milk after 1, 2, 3, 4 and 5 days of fermentation, respectively.

Table S1. List of amino acid modifications (PTMs) of bovine κ-CN according to the online database UniProtKB (Last access on 24.05.2022)

Table S2. List of peptides obtained after the in-silico tryptic digestion of κ-CN using the PeptideCutter tool of UniprotKB. The information on the sequence of the primary chains was collected from the online database UniProtKB (Accession number: P02668|CASK_BOVIN). Red and green colors represent glycosylated and phosphorylated amino acid residues, respectively.

Table S3. List of peptides obtained after the in-silico GluC-E digestion (Ambi buffer) of k-casein. The information on the sequence of the primary chains was collected from the online database UniProtKB (Accession number: P02668|CASK_BOVIN). Red and green colors represent glycosylated and phosphorylated amino acid residues, respectively

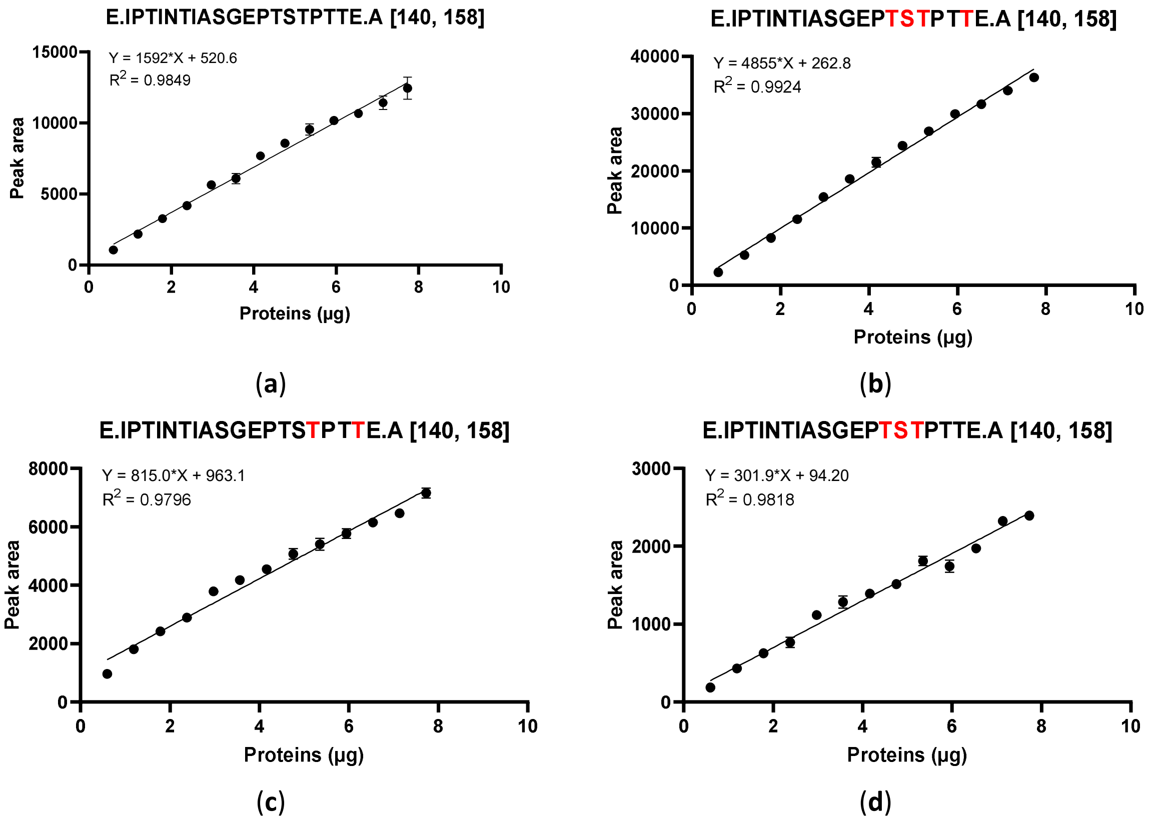

Figure S1. Linearity of the peptide (a) E.IPTINTIASGEPTSTPTTE.A [140, 158] and it modified forms (b) IPTINTIASGEPTSTPTTE [140, 158], (c) IPTINTIASGEPTSTPTTE [140, 158] and (d) IPTINTIASGEPTSTPTTE [140, 158] using the developed LC-MS/MS method. Modified serine and threonine residues are underlined. Red and blue colors indicate glycosylation and phosphorylation modifications, respectively.

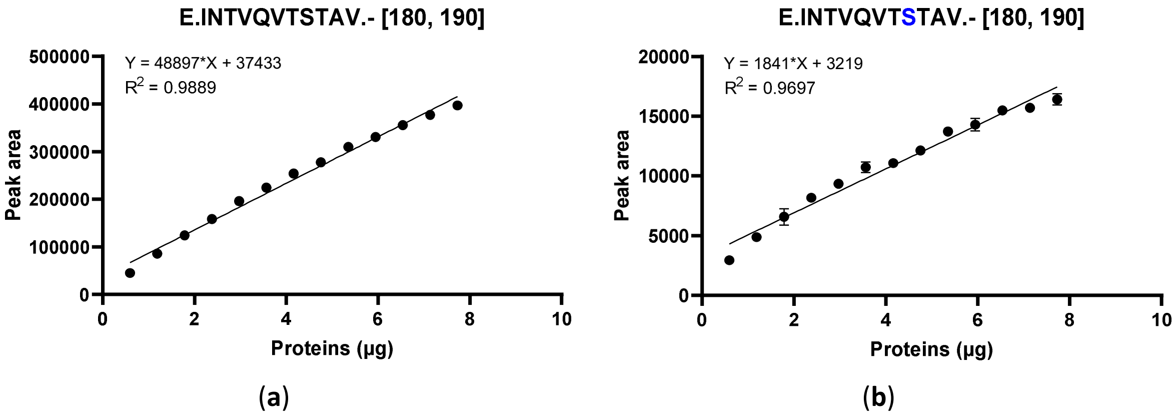

Figure S2. Linearity of the peptide (a) E.INTVQVTSTAV [180, 190] and it modified form (b) INTVQVTSTAV [180, 190] using the developed LC-MS/MS method. Modified serine and threonine residues are underlined. Red and blue colors indicate glycosylation and phosphorylation modifications, respectively.

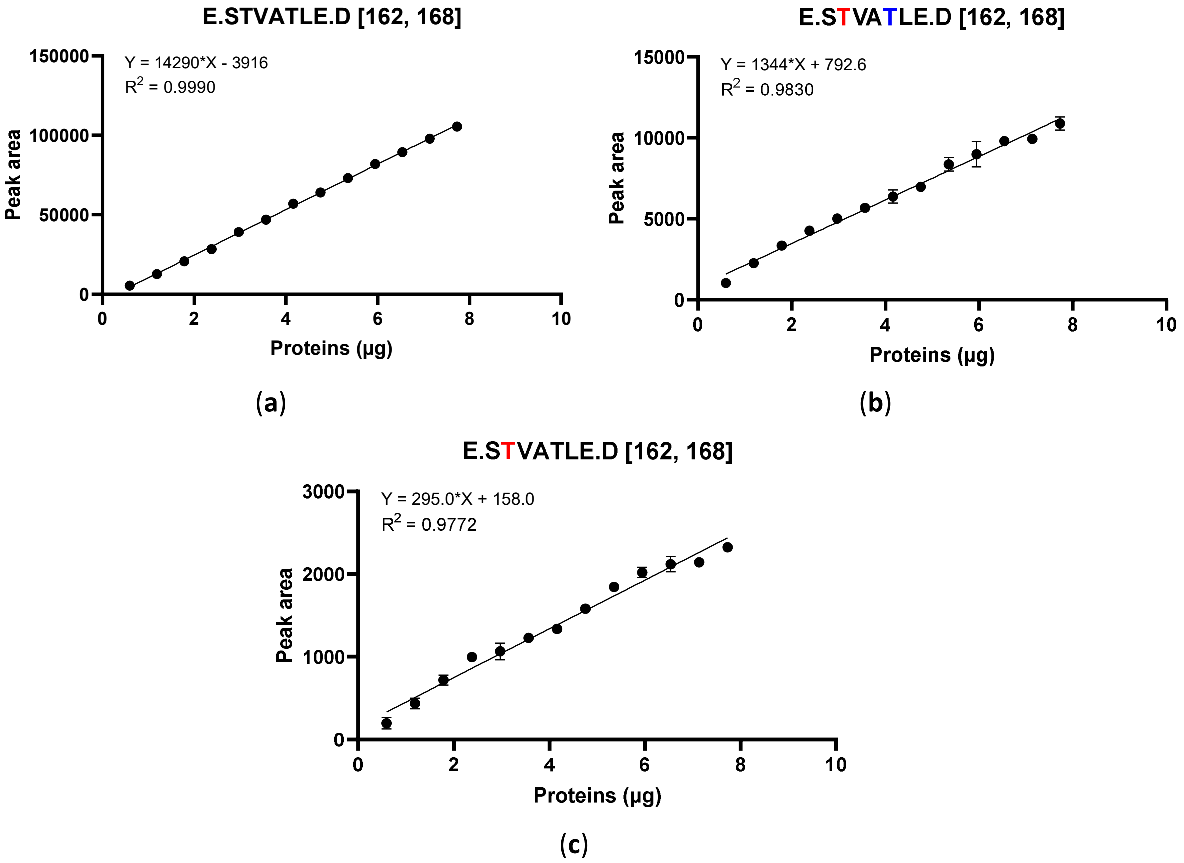

Figure S3. Linearity of the peptide (a) E.STVATLE.D [162, 168] and it modified forms (b) S<u>T</u>VA<u>T</u>LE [162, 168] and (c) S<u>T</u>VATLE [162, 168] using the developed LC-MS/MS method. Modified serine and threonine residues are <u>underlined</u>. Red and blue colors indicate glycosylation and phosphorylation modifications, respectively.

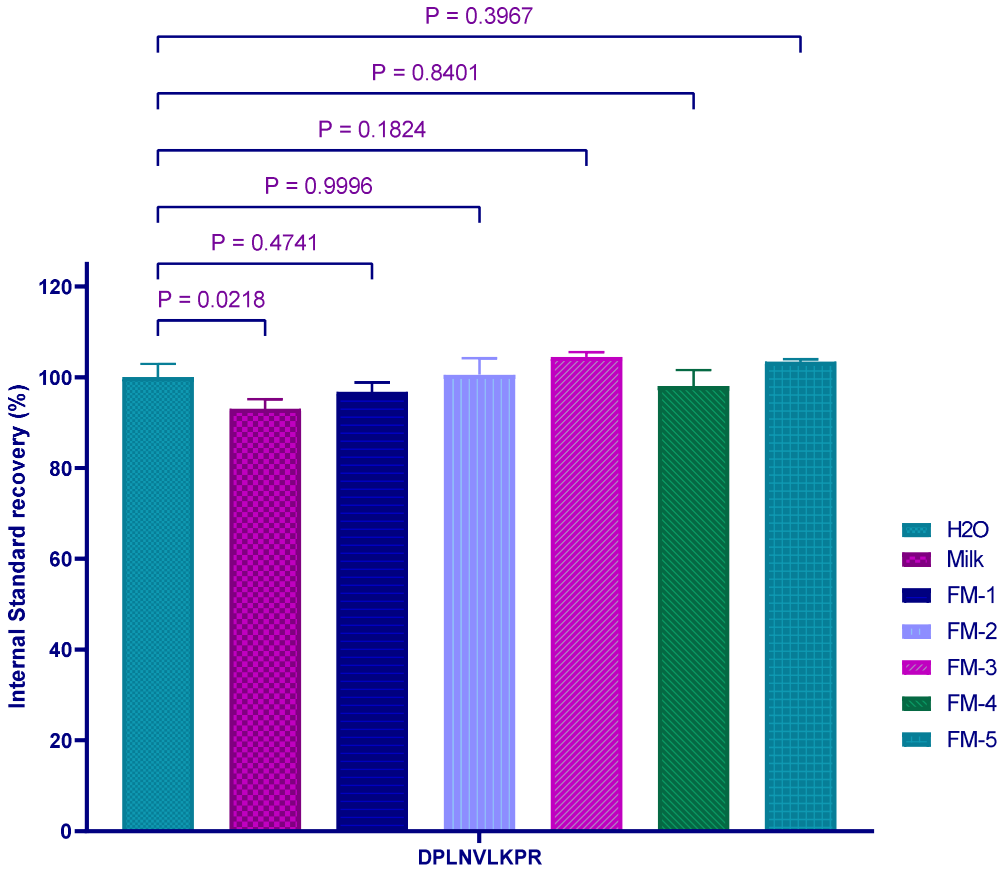

Figure S4. Recovery of the injected internal standards DPLNV(d8)LKPR in the different milk and fermented milk matrixes. The experiments were performed in triplicate and the results are expressed as percent (%) comparing to the internal standard in distilled water (n=3). FM-1, FM-2, FM-3, FM-4 and FM-5 are the fermented milk after 1, 2, 3, 4 and 5 days of fermentation, respectively.

Table S1. List of amino acid modifications (PTMs) of bovine κ-CN according to the online database UniProtKB (Last access on 24.05.2022)

Feature key	Position(s)	Description Actions
Modified residue	22	Pyrrolidone carboxylic acid1 Publication
Disulfide bond	32 ↔ 109	
Disulfide bond	32	Interchain (with C-109); in linked form
Disulfide bond	109	Interchain (with C-32); in linked form
Glycosylation	142	O-linked (GalNAc...) threonine3 Publications
Modified residue	148	Phosphoserine1 Publication
Glycosylation	152	O-linked (GalNAc...) threonine3 Publications
Glycosylation	153	O-linked (GalNAc...) serine1 Publication
Glycosylation	154	O-linked (GalNAc...) threonine3 Publications
Glycosylation	157	O-linked (GalNAc...) threonine3 Publications
Glycosylation	163	O-linked (GalNAc...) threonine3 Publications
Modified residue	166	Phosphothreonine1 Publication
Modified residue	170	Phosphoserine; alternate2 Publications
Glycosylation	170	O-linked (GalNAc...) serine; alternate1 Publication
Glycosylation	186	O-linked (GalNAc...) threonine; partial3 Publications
Modified residue	187	Phosphoserine by similarity

Table S2. List of peptides obtained after the in-silico tryptic digestion of κ-CN using the PeptideCutter tool of UniprotKB. The information on the sequence of the primary chains was collected from the online database UniProtKB (Accession number: P02668|CASK_BOVIN). Red and green colors represent glycosylated and phosphorylated amino acid residues, respectively.

No	Position of cleavage site	Resulting peptide sequence	Peptide length [aa]	Peptide mass [units]
1	3	MMK	3	408.6
2	31	SFFLVVTILALTLPFLGAQEQNQEQPIR	28	3173.7
3	34	CEK	3	378.4
4	37	DER	3	418.4
5	42	FFSDK	5	642.7
6	45	IAK	3	330.4
7	55	YIPIQYVLSR	10	1251.5
8	89	YPSYGLNYYQQKPVALINNQFLPYPYYAKPAAVR	34	4011.6
9	107	SPAQILQWQVLSNTVPAK	18	1980.3
10	118	SCQAQPTTMAR	11	1193.4
11	132	HPHPHLSFMAIPPK	14	1608.9
12	133	K	1	146.2
13	137	NQDK	4	503.5
14	-	TEIPTINTIASGEPTSTPTTEAVESTVATLEDSPEVIESPPEINTVQVTSTAV	53	5455.9

```
                                             Tryps
                                          Tryps  |
                                     Tryps    |  |
                                  Tryps  |    |  |
   Tryps                       Tryps  |  |    |  |     Tryps

       |                           |  |  |    |  |         |
1    MMKSFFLVVTILALTLPFLGAQEQNQEQPIRCEKDERFFSDKIAKYIPIQYVLSRYPSYG
     ---------+---------+---------+---------+---------+---------+   60


                             Tryps             Tryps      Tryps

                                 |                 |          |
61   LNYYQQKPVALINNQFLPYPYYAKPAAVRSPAQILQWQVLSNTVPAKSCQAQPTTMARHP
     ---------+---------+---------+---------+---------+---------+   120


                 Tryps
             Tryps   |
            Tryps|   |

                ||   |
121  HPHLSFMAIPPKKNQDKTEIPTINTIASGEPTSTPTTEAVESTVATLEDSPEVIESPPEI
     ---------+---------+---------+---------+---------+---------+   180


181  NTVQVTSTAV
     ---------+   190
```

Table S3. List of peptides obtained after the in-silico GluC-E digestion (Ambi buffer) of κ-CN. The information on the sequence of the primary chains was collected from the online database UniProtKB (Accession number: P02668|CASK_BOVIN). Red and green colors represent glycosylated and phosphorylated amino acid residues, respectively.

No	Position of cleavage site	Resulting peptide sequence	Peptide length [aa]	Peptide mass [units]
1	23	MMKSFFLVVTILALTLPFLGAQE	23	2570.2
2	27	QNQE	4	517.5
3	33	QPIRCE	6	744.9
4	36	KDE	3	390.4
5	139	RFFSDKIAKYIPIQYVLSRYPSYGLNYYQQKPVALINNQFLPYPYYAKPAAVRSP AQILQWQVLSNTVPAKSCQAQPTTMARHPHPHLSFMAIPPKKNQDKTE	103	11910.8
6	150	IPTINTIASGEPTSTPTTE	11	1948,1
8	161	AVE	3	317.3
9	168	STVATLE	7	719.8
10	172	DSPE	4	446.4
11	175	VIE	3	359.4
12	179	SPPE	4	428.4
13	-	INTVQVTSTAV	11	1132.3

```
                                   Glu
                         Glu Glu   Glu  |

                          |   |     |  |
      MMKSFFLVVTILALTLPFLGAQEQNQEQPIRCEKDERFFSDKIAKYIPIQYVLSRYPSYG
   1  ---------+---------+---------+---------+---------+---------+   60


      LNYYQQKPVALINNQFLPYPYYAKPAAVRSPAQILQWQVLSNTVPAKSCQAQPTTMARHP
  61  ---------+---------+---------+---------+---------+---------+   120


                                         Glu           Glu
                  Glu        Glu     Glu  |      Glu Glu  |  Glu

                    |          |       |  |        |   |  |   |
      HPHLSFMAIPPKKNQDKTEIPTINTIASGEPTSTPTTEAVESTVATLEDSPEVIESPPEI
 121  ---------+---------+---------+---------+---------+---------+   180


      NTVQVTSTAV
 181  ---------+   190
```
